# Choosing efficient actions: Deciding where to walk

**DOI:** 10.1371/journal.pone.0219729

**Published:** 2019-09-26

**Authors:** Sally A. Linkenauger, Veronica Weser, Dennis R. Proffitt

**Affiliations:** 1 Department of Psychology, Lancaster University, Lancaster, United Kingdom; 2 Department of Psychology, University of Virginia, Charlottesville, VA, United States of America; University of Exeter, UNITED KINGDOM

## Abstract

Humans evolved to be endurance animals. Our ancestors were persistence hunters; they would chase animals, including gazelles, until they ran them into exhaustion. Put simply, people evolved in an ecological niche that selected for endurance and efficiency of locomotion. To locomote to any destination, one could take countless different paths, each requiring different amounts of energy. Because the ground is typically not flat or homogeneous, the straight direct path is often not the most energetically efficient. For hills below 14°, the direct straight path up the hill is the most energetically efficient. However, for hills above 14°, walkers would minimize their absolute energy expenditure by taking a zigzagged path so that their gradient of ascension is 14° [1]. In three experiments, we assessed the degree to which people make bioenergetically efficient decisions about locomotion through path selection. In Experiment 1, people were immersed into a virtual environment and adjusted the angle of ascension of a virtual path up hills of various gradients so that when taking the path, they would expend the least amount of energy when they reached the top. The second experiment was of a similar design, but was conducted in the real word. In the last experiment, in a virtual environment, participants choose between two paths up hills of various gradient, where these paths varied in the energy required for ascent. Participants made these judgements both before and after motor experience with gradient climbing on an incline trainer. For steep hills, we found that people choose much straighter paths over the bioenergetically optimal zigzagged paths. Motor experience did lead to higher probability for choosing optimal paths for steep hills, but lead to less optimal paths for shallower ones. These results show clearly that individuals show a straight path bias when deciding how to ascend hills.

## Introduction

The most fundamental economic decisions that people make concern the costs and benefits of expending energy. For all organisms, energy is a limited resource. Yet, everything that we do, from running a marathon to reading this sentence, results in some form of energetic expenditure. In our day-to-day lives, our energetic resources are unlikely to be in danger of depletion as our energetic output is typically low and the available energetic replenishment in our environment is copious. Yet in several contexts, our ability to conserve our available energy is crucial to our survival. Consider backpackers lost in the woods around the English Lake District, soldiers traversing across a hostile landscape, or injured and/or chronic pain patients and older people attempting to fulfill their daily responsibilities. For each of these groups, taking the route of least overall energetic expenditure to one’s destination could be the difference between safety and danger, freedom and imprisonment, or autonomy and dependence.

Human’s bodies, more than most other mammalian species, evolved for physical endurance. Humans have evolved enhanced endocrine systems and morphological adaptations to complement long distance locomotion [2]. In fact, for distances of and over 20 miles, on a hot day, humans are one of the fastest land mammals on earth. Presumably, this energetic advantage allowed humans to prosper by running prey to exhaustion in harsh habitats with fierce competition from other predators [3]. Put simply, we evolved in an environment where endurance and efficiency of locomotion was indispensable to our survival, which made the conservation of energy of paramount importance. Because landscape topography is rarely flat and varies in steepness, the straightest path is often not the most energetically conservative route. Thus, humans must regulate their energetic expenditure across terrains of different acclivities. Optimizing energy expenditure in such conditions requires humans to determine the most energetically efficient route to their desired destination. However, little is known about this fundamental economic choice process, human’s perceptual sensitivity to the energetic costs associated with locomotion on alternative paths across terrains of various inclines. Here, we conducted three studies in which we assessed perceptual sensitivity to bioenergetic costs of different routes in slant ascension by having individuals discriminate between paths based on their bioenergetic costs.

**Fig 1:**
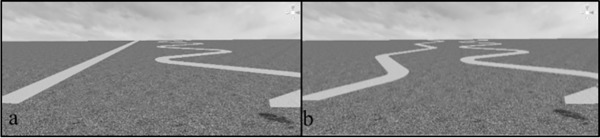
Depiction of two paths up a geographical slant that differ in steepness and length. The path in Panel A is shorter but steeper, while the path in Panel B is longer and shallower.

Much is known about the bioenergetic costs of locomotion across different types of terrains, and specifically, the bioenergetic costs associated with humans ascending and descending geographical slants have been well documented [1,4,5,6,7]. As people locomote directly up steeper gradients, they expend more energy; however, they also traverse the shortest distance to reach the given height. To travel at a shallower gradient and, therefore, expend less energy with respect to gradient, people could take a zigzagged path up the slope. However, they will traverse a longer distance to reach the same height, which requires more energy with respect to path length. As a result, there is an energetic tradeoff between path steepness and path length in terms of the overall energy expended traversing the hill. See Fig 1. The left path (Panel A) is shorter than the right path (Panel B), which means that less energy will be spent with respect to path length; however, the left path ascends at a steeper gradient than the right path, which means more energy will be spent with respect to path steepness.

**Fig 2:**
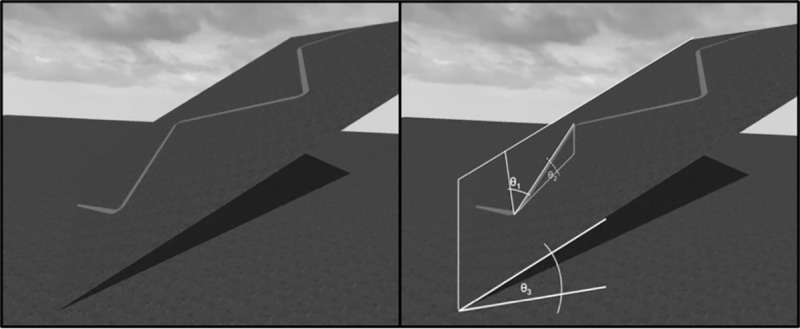
Panel A: A geographical slant with a slope of 30°. Panel B: Given the slope of hill and the desired ascension gradient, one can calculate the angle of deviation of the path from the straight path (θ_1_) via the equation: $\theta_{1}={sin}^{-1}(\frac{{\sin\theta }_{2}}{{\sin\theta }_{3}})$, where θ_2_ is desired path gradient and θ_3_ is the slope of the hill.

When ascending (or descending) any given gradient, the most energetically economical gradient path is the one in which the product of the energy expended with respect to steepness and path length is minimized (see [1], for more detail). For geographical slants below 14°, the most energetically economical path is the direct, straight path. For geographical slants above 14°, the most energetically economical path is at a zigzag in which the gradient of ascension (or descension) is 14° [1]. Consider a 30° slope (θ_3_, see Fig 2). A walker could take a countless number of paths to ascend the slope. For example, she could take a straight path up the slope, and thereby, ascend at a gradient of 30°; or she could deviate from a straight path by 62° to the right (or 28° from the horizontal, θ_1_), traversing the slope in a zigzagged path, and thereby ascend at a gradient of 14° (θ_2_). By taking the latter path, the walkers will have reached her destination by expending the minimal amount of energy. See [8,9, 10] for similar work on routes and bioenergetics.

Although the optimal path in hill ascent and descent can be calculated, as of yet, it is unknown whether humans are sensitive to the energetic costs of various alternative paths. For example, in the absence of sophisticated equipment or advanced knowledge of physiology, would our aforementioned walker be able to determine that a 14° gradient of ascension (zigzagged path) up a 30° hill is the most energetically economical path? If humans are sensitive to the energetic costs of various alternative paths, the precision of this sensitivity is also unclear. Would our hiker know to take a zigzagged path at exactly 14° or would she only be sensitive an approximate range of zigzagged paths around 14°?

The only indirect evidence that suggests that individuals could potentially be sensitive to the optimal paths up (and down) hills comes from looking at pre-existing mountain paths. After determining the most energetically optimal incline to which hills should be ascended and descended, Minetti [1] assessed the gradient of ascension of randomly selected mountain paths in the Italian Alps and the Himalayan mountains for slopes steeper than 14°. Of the 31 paths analyzed, the mean path gradient was 15.58° (SD = 4.30°). Such a coincidence of the mountain paths being so close to the most energetically economical incline is unlikely to have occurred by chance. However, these mountain paths have evolved over an indefinite number of years by numerous travelers many of whom have likely ascended and descended the same slope several times. Hence, these path gradients could be a result of human’s sensitivity to the energetic costs of different routes, but could also be explained by trial and error from several attempts or even humans following paths previously taken by other animals. Consequently, these results cannot be generalized to human perception, especially on an individual basis.

However, there is good reason to believe that humans should have at least a rudimentary sensitivity to the energetic costs of different gradients of ascent (descent). Indeed, previous research has shown that humans are extremely sensitive to their energetic expenditure in other tasks as evidenced by fast rates of motor adaptations to the most energetically optimal method of performance [11, 12]. Specifically, while performing motor tasks, humans tend to change their motor behavior over time to “self-optimize”, which according to Sparrow & Newell [13] is the “natural adoption of a movement pattern that minimizes movement expenditure” (p. 179). For example, humans automatically adjust their gait when locomoting at different speeds to achieve metabolic efficiency [6, 14]. Also, when running at a fixed stride rate and speed, individuals naturally adopt the most energetically efficient stride length [15]. Even when performing novel motor tasks, humans quickly adjust to minimize energetic expenditure. Such as, when crawling on a treadmill at a constant speed, humans adjust their four-limbed gait to achieve optimal metabolic efficiency across different gradients [16]. In addition to novel tasks, there is some evidence that experience with a behaviour does actually lead to faster and higher levels of self-optimization; those without experience tend to be more variable and take longer to achieve a lower degree of self-optimization [17, 18].

Although it is fascinating that our motor systems are capable of automatically adjusting to optimize motor performance, it is unlikely that individuals are aware and consciously making decisions about slight biomechanical adjustments, which optimize their energetic efficiency. Hence, it is unclear as to whether this self-optimization can be generalized to when individuals are making explicit decisions about route choice and consequently, people may be unable to select the most bioenergetically efficient route.

Indeed, most people have little or no experience climbing hills above 15° as we rarely encounter them in our environment. For example, the steepest road in the UK, Hardknott Pass, Cumbria, has an incline of 18°. The steepest paved road in the world, Baldwin Street, NZ, is only 19° [19]. Typically, commonly locomoted paths with inclines steeper than 15° are accompanied by staircases, which reduce the kinematic and energetic costs associated with climbing the slope. In fact, when stairs and slopes are of the same steepness, the bioenergetic costs of climbing stairs always exceeds the costs of climbing slopes [20]. It is likely that people have had little or no experience determining the optimal path for hills above 15°, because such steep inclines would have rarely or never been encountered. Instead, people may adopt a heuristic to always take the straight path up a hill, because for hills below 15°, the straight path is optimal. Hence, this heuristic would be a satisficing solution similar to those seen in decision making processes [21]. If this is the case, then individuals should be more likely to take a straight path even when a zigzagged path is more energetically efficient.

We addressed this question by conducting several experiments in which individuals judged the bioenergetic costs associated with different routes in hill ascension by determining the bioenergetically optimal path using the psychophysical methods of adjustment and constant stimuli. In order to present hills of various steepness with paths of different ascension gradients, two of the experiments were conducted in virtual reality. To assure generalizability of our results in virtual reality, we also conducted a study in the real world.

## Experiment 1: Path adjustment in virtual reality

To assess human’s ability to identify the bioenergetically optimal path when ascending hills, we immersed participants in a virtual environment in which they stood at the foot of a hill. They were instructed to adjust the ascension gradient of a pathway so that the pathway required the least amount of energy to ascend to the top of the hill.

### Methods

#### Participants

Twelve individuals (5 female) were recruited to participate in the experiment via opportunity sampling from Lancaster University. All participants had normal or corrected-to-normal vision and had no morphological abnormalities. All participants provided informed consent and the study was approved by the Lancaster University Ethic Committee.

#### Stimuli and apparatus

The virtual environment was presented in an Oculus Rift DK2 head-mounted display (HMD) which provided stereopsis. The virtual environment and experimental program was programmed using Unity3D gaming engine. The participants provided responses using a standard Xbox controller. The environment consisted of a grassy hill. On the hill, we positioned a brick path as well as a park bench to provide cues to size and depth. The textures of the grass and path were consistent to provide participants with cues due to texture gradient. The program consisted of 22 trials of 11 hills (0–55° in steps of 5°) each of which were presented twice. Participants could adjust the degree of the path’s zig-zag on the hill using the rear top triggers on the Xbox controller. The left trigger decreased the degree of zig-zag, and the right trigger increased the zig-zag. When the “a” button was pressed, the response was recorded and the program proceeded to the next trial. In half the trials, the path starting position was straight, and in half, the path starting position was a 45° deviation from vertical (zigzag).

#### Procedure

Prior to donning the HMD, participants held Xbox controller and were instructed how to perform the task. Specifically, participants were instructed to view the hill and respond as to whether they thought themselves capable of walking up the hill without climbing (without using their hands). If they responded that they could ascend the hill, they were to use the triggers to adjust the degree of zigzag of the path so that the path was the most energetically efficient. Participants did not make estimates for paths that they responded that could not ascend. To increase understanding, participants were told that energetically efficient could also be characterized as the easiest path upon which they would have expended the least amount of energy when having reached the top of the hill. When they were satisfied with their path estimate, they were told to press the “a” button to progress to the next trials. After completing all the trials, participants took off the HMD and completed a questionnaire that assessed physical fitness, exercise practices, and frequency of walking and climbing hills.

### Results

For one of the participants, we experienced technical difficulties with the equipment, and as a consequence, their data was not included in the analysis. On average, participants estimated the maximum hill up which they could ascend was 36.59° (*SD* = 9.76°). We assessed accuracy of responses by subtracting the energetically optimal angular deviation from the vertical path from their estimates of deviation from the vertical path for each hill to create an error score. Hence, positive error scores signal that participants had a smaller gradient of ascension than optimal (too much zigzag). Negative scores signal that participants had a larger gradient of ascension than optimal (not enough zigzag).

**Fig 3:**
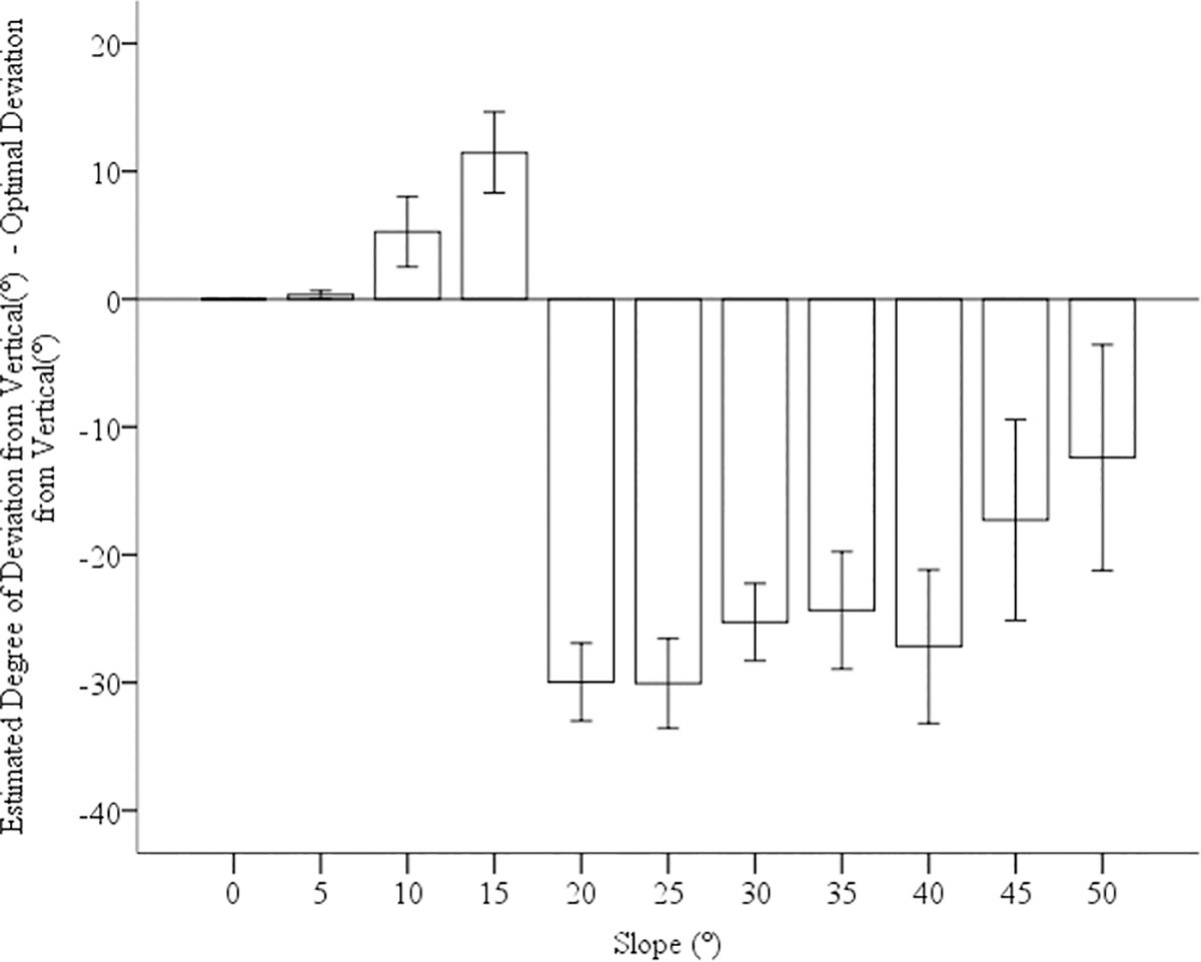
Error scores for each slope. Error bars represent 95% CIs.

To assess whether error differed as a function of hill slant, we conducted a univariate analysis of variance (ANOVA) with participant as a random factor, slope as fixed factor, and error score as the dependent measure. Slope was significant with higher slopes being more negative than shallower slopes, *F*(10, 77.17) = 27.79, *p*<0.001, *ŋ*_*p*_^*2*^ = .78. This difference appears to be driven by slopes above and below 15°, see Fig 3. Consequently, we conducted two more of the same ANOVAs for hills above and below 15°. For hills above 15°, we found no significant difference in error scores as a function of slope, *p* = .38. The average participant error for hills above 15° was -26.67° (*SE* = 2.86°). Using a one-sample t-test, we tested the average of participants’ errors for hills above 15° against 0 (accuracy). We found that participants’ errors were significantly less than zero, *t*(10) = 9.31, *p*< 0.001, *d* = 2.81. This finding suggests that participants opted for paths of a higher gradient of ascension or less zigzag than optimal.

**Fig 4:**
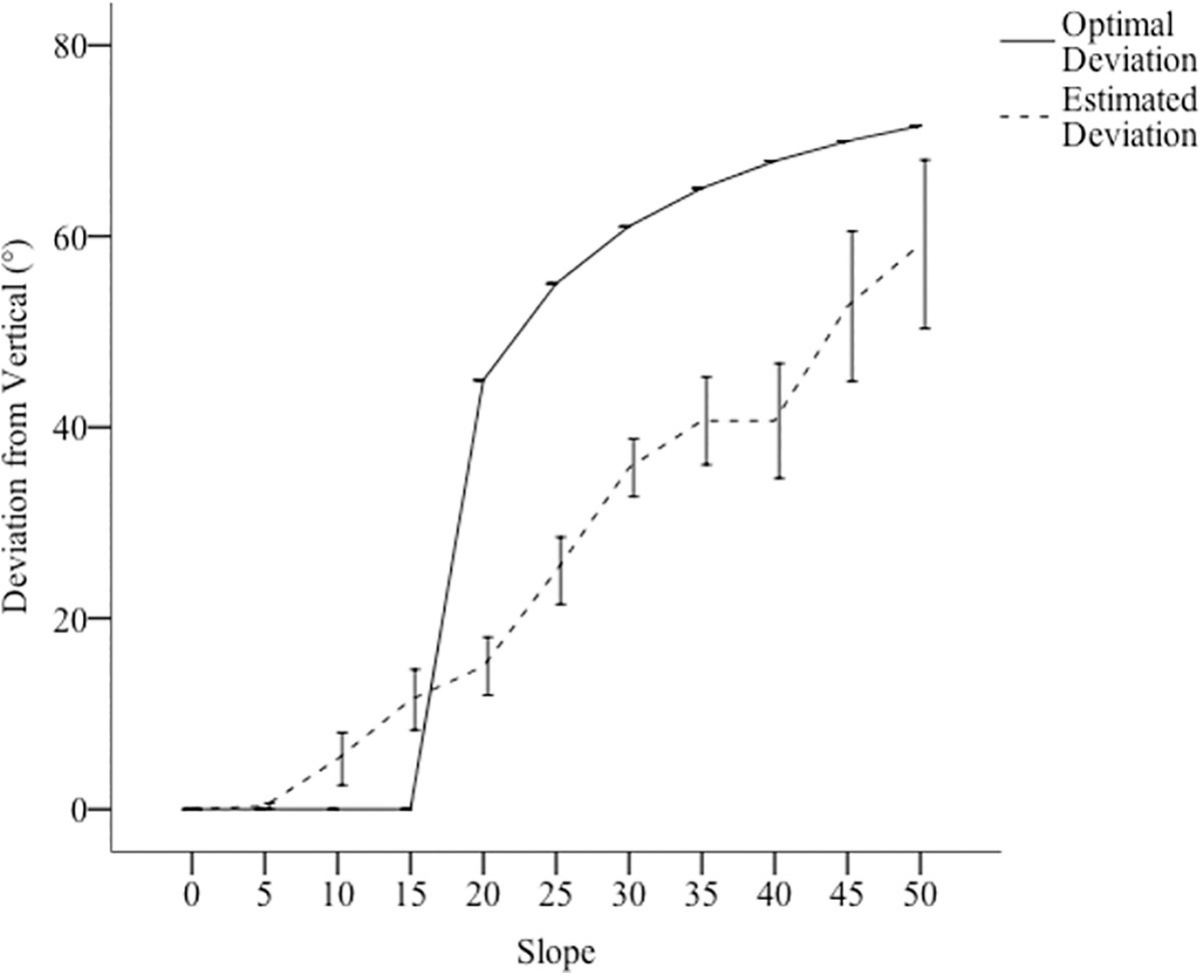
Participants estimations of their deviation from vertical in comparison to optimal. Error bars represent +/- 1 SEM.

For hills below 15°, error estimates did significantly differ with respect to slope, *F*(10, 30.57) = 5.20, *p =* .005, *ŋ*_*p*_^*2*^ = .34. Using a one-sample t-test, we tested the average of participants’ errors for hills below 15° against 0 (accuracy). We found that participants’ errors, *M* = 4.31, *SE* = 1.34, were significantly greater than zero, *t*(10) = 3.23, *p* = 0.001, *d* = .97. Post-hoc comparisons (Bonferroni corrections) showed that the 15° hill (*M* = 11.47°, *SE* = 1.80°,) had more positive error than the 0° (*M* = 0.0°, *SE* = 1.88°, *p* < .001), 5° (*M* = 0.32°, *SE* = 1.88°, *p* = .001) but not the 10°(*M* = 5.27, *SE* = 1.80, *p =* .12), *ps* < .05. There were no significant differences between the errors in the 0°, 5°, and 10° hills, *ps* > .28. This finding suggests that for 15° hills, participants chose paths of a lower gradient of ascension or more zigzag than optimal, see Fig 4. We did not find any relationship between the questionnaire responses and route choices across any of these experiments.

## Experiment 2: Path adjustment in the real world

To ensure our results in virtual reality were generalizable to real environments, we conducted a similar design as in Experiment 1, but in the real world. Individuals made judgements about their route choice on a steep hill on campus. Participants also made judgements of hill steepness. With respect to geographical slant perception, a large field of research has investigated how individuals perceive the steepness of hills. In general, people drastically overestimate geographical slant [22]. However, individual differences in slant perception vary depending on the energetic state of the perceiver. Those who are out of shape, encumbered by a heavy object, fatigued, or ageing and elderly perceived hills to be steeper than those who are in shape, energized and young. Hence, we see the steepness of a given hill with respect to the energetic costs associated with ascending the hill [23]. Hence, here, were also interested if individuals’ perceptions of steepness were related to route choice.

### Methods

#### Participants

Twenty-six University of Virginia students (8 male, mean ± SD age: 19.42 ± 0.99 years, rage 18–22) participated. All participants provided informed consent and the study was approved by the University of Virginia Ethics Committee. All had normal or corrected-to-normal vision. They were naive to the purpose of the experiment.

#### Stimuli and apparatus

A hill on the grounds of the University of Virginia was used in the study. The hill was always viewed from the front, in daylight. Participants stood at the base of the hill, with their view of the hill unobstructed. The average inclination of the hill was 26º as measured by a Suunto clinometer having an accuracy of 0.5".

**Fig 5:**
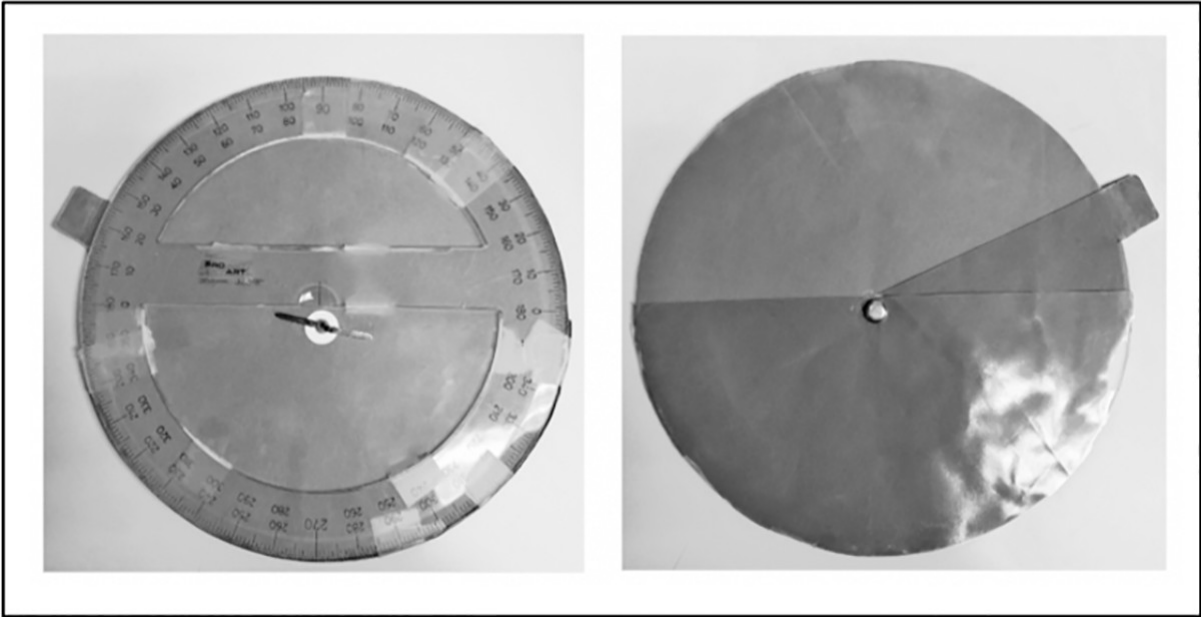
Panel A: the experimenter view of the visual disk with the protractor. Panel B: the adjustable hill seen by participants. The slop of the hill was changed by moving the green tab so that the resulting green segment best represented a cross-section of the hill.

The visual judgments of hill steepness were made by using a disk that consisted of an adjustable angle representing the cross-section of the inclination of a hill affixed to a protractor. See Fig 5.

When making their visual judgments, participants were asked to adjust the disk by moving the green tab either closer to or further away from the green bottom half of the disk depicting the horizon or a 0° slant, with the aim of making a cross-section that best represented the angle of inclination of the hill. The protractor (in Panel A) was never seen by the participant, but it allowed the experimenter to determine the angle to which the participant set the cross-section. The participants usually held the disk approximately perpendicular to their line of sight while observing the hill head-on.

The participants reported their haptic judgments by using a tilt board with a flat palm rest; the tilt of the board could be adjusted upward or downward to match the slope of the hill. The tilt board also had a digital protractor on its side which allowed the experimenter to determine the angle the subject chose. The tilt board itself was mounted on a tripod whose height could be adjusted to about waist level for each individual participant. While reporting their haptic judgments, participants were asked to match the tilt board to the slope of the hill before them using their dominant hand. The tilt board was out of the participant’s view and they were not permitted to look at their hand while making the adjustments.

#### Procedure

The participants viewed the hills binocularly from the front while standing on an “X” clearly marked in white paint at the base of the hill. Participants were instructed to look directly ahead and to refrain from looking at the hill sideways. Participants made three separate judgements of the angle of inclination of the hill with respect to the horizontal: visual, haptic and a novel angle of ascent task for two different distances up the hill (331cm and 220cm) in a randomly assigned order.

To determine the ideal angle of ascent, a second experimenter stood directly in front of the participant at either the near (220 cm) or far (331 cm) distance up the hill. The participant was instructed to pick a path up the hill that would leave him or her with the most energy upon reaching the top if climbing at a comfortable pace. Upon selecting that angle, they were told to instruct the experimenter to move to the left or to the right (counter balanced across near and far distances) until the line between the participant and the experimenter matched the ideal angle of ascent. Once the experimenter on the hill was in place, the participant climbed the hill in a straight line at the angle they chose. Upon reaching the second experimenter, the participant was asked if the chosen path was too steep, too shallow or just right. The research assistants running the study had no expectations about how the participants ought to behave, nor were they aware of the bioenergetically optimal path. The response was recorded.

The participant then descended the hill at the same angle they ascended while holding the zero-end of the tape measurer. Once the participant reached the “X” at the base of the hill, the experimenter on the hill recorded the distance. This distance was later used to calculate the angle the participant selected using arccosine of the predetermined distance over the distance the participant climbed. Participants never saw the distance traveled, nor did they see the experimenter climb up the hill. If the participant judged the path climbed as too steep or too shallow, the procedure was repeated until the angle was deemed appropriate for the hill. No feedback regarding the choice of path was given. After the participant finished all 3 judgment tasks, they completed a questionnaire that assessed physical fitness, exercise practices, and frequency of walking and climbing hills.

### Results

As in Experiment 1, we calculated error scores by subtracting the optimal deviation from the vertical path from participants’ estimates of the angular deviation from the vertical path for both the near and far task. We calculated that the energetically optimal angular deviation from vertical for this hill is 56.81°.

To investigate participants’ accuracy in the task, we conducted two one-sample t-tests comparing participants’ error scores in the near and far condition to 0° (perfect accuracy). For the near task, error scores were significantly less than 0, *M* = -19.87°, *SE* = 2.32°, *t*(25) = 8.56, *p* < .001, *d* = 1.68.

**Fig 6:**
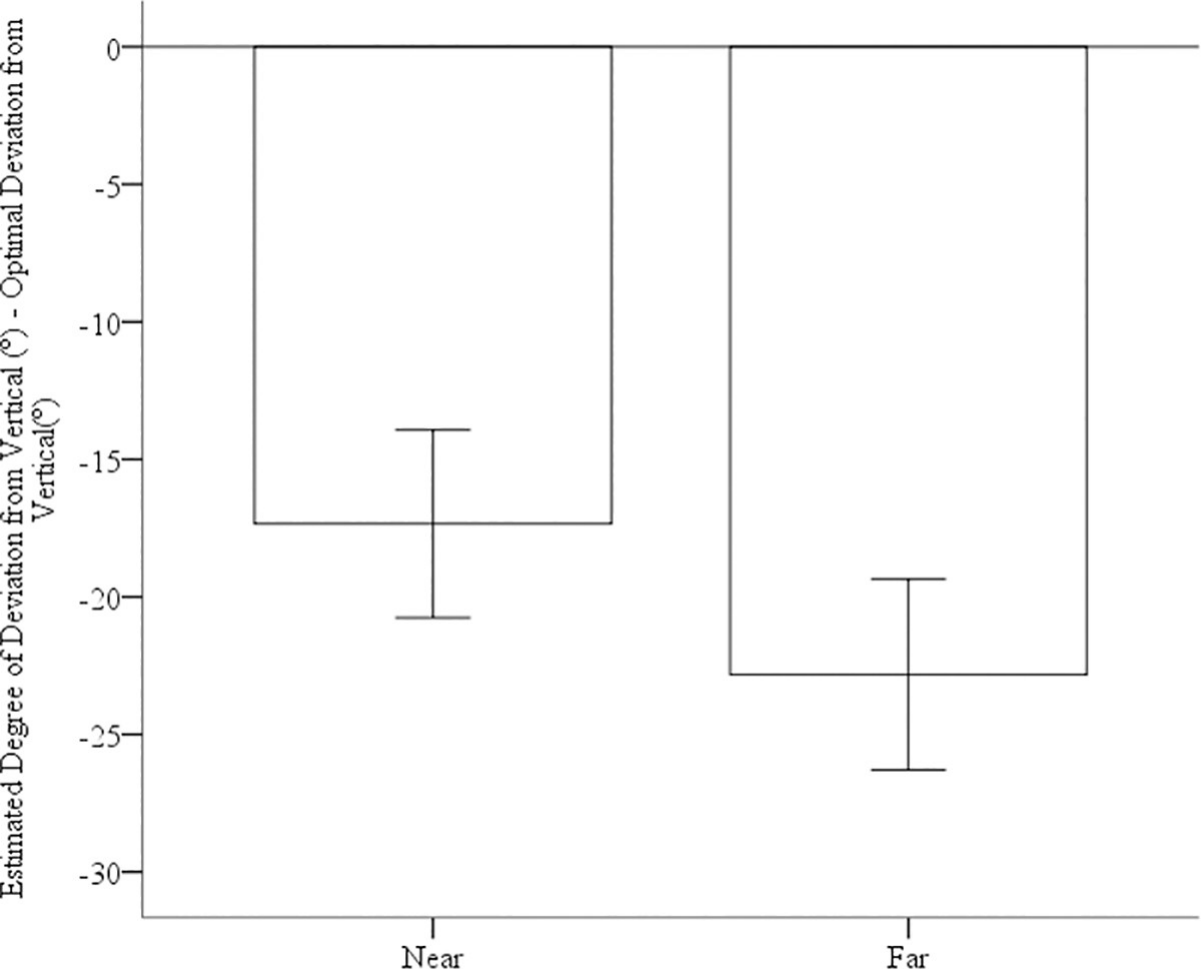
Error scores for near and far estimates of route choice. Error bars represent 95% CIs.

For the far task, error scores were also significantly less than 0, *M* = -23.09°, *SE* = 1.55°, *t*(25) = 14.85, *p<* 0.001, *d =* 2.92. To compare near and far estimates, we conducted a paired samples t-test, with near versus far as the within-subjects variable and error scores as the dependent measure. We found no significant different difference between the near and far estimates, *t*(25) = 1.47, *p* = 0.16, two-tailed test, see Fig 6.

**Fig 7:**
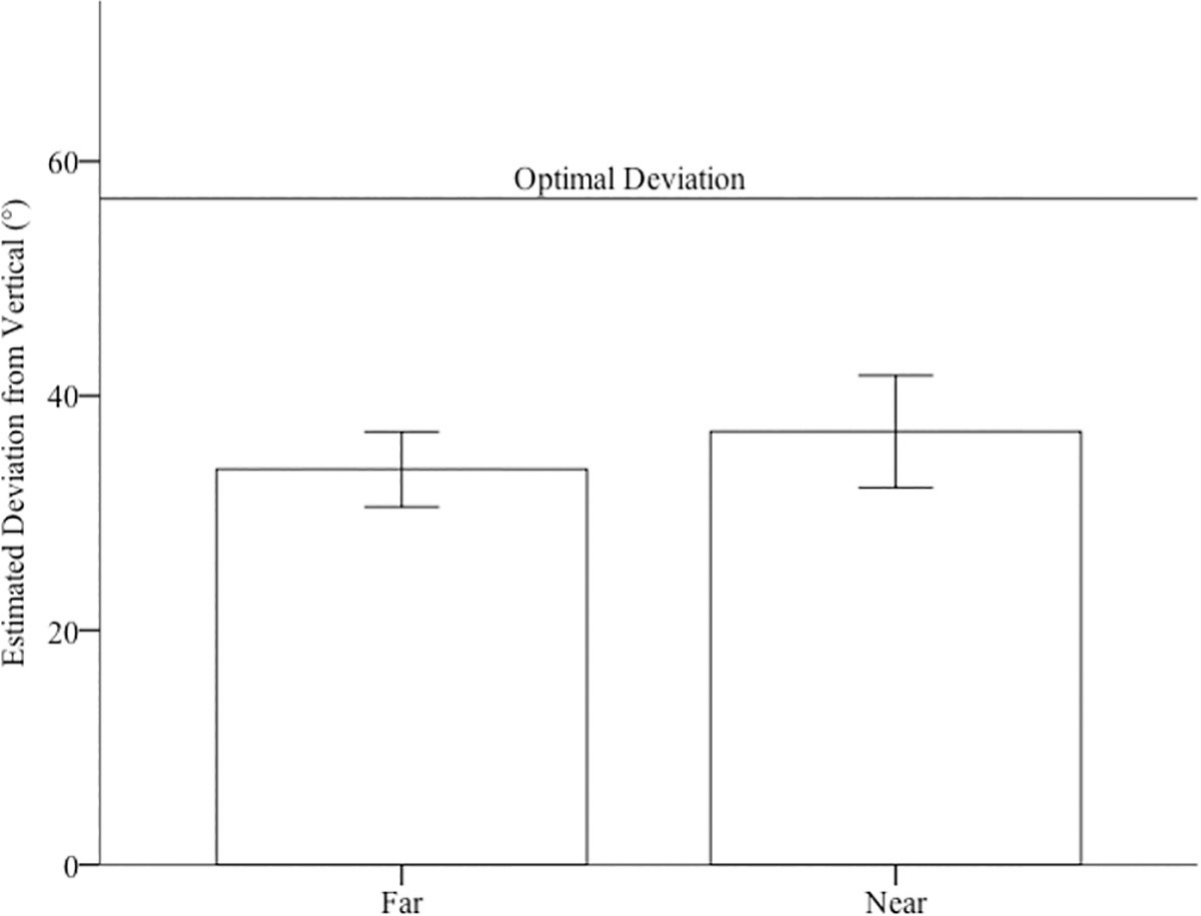
Estimates of the path’s deviation from vertical for both the near and far path. Error bars represent 95% CIs.

As found in Experiment 1, participants opted for paths whose gradient of ascension was steeper than optimal. Put simply, individual chose paths than did not have a high enough deviation from vertical. However, the average participant error for the 25° path in Experiment 1 was 32°, so participants’ performance was better than in this experiment, see Fig 7. However, the tasks were a bit different, and additionally, the participants in each experiment were culturally different. The important point here to note is that participants in both the virtual reality and real world studies both opted for paths whose gradient of ascensions were steeper than optimal and to a relatively similar degree.

**Fig 8:**
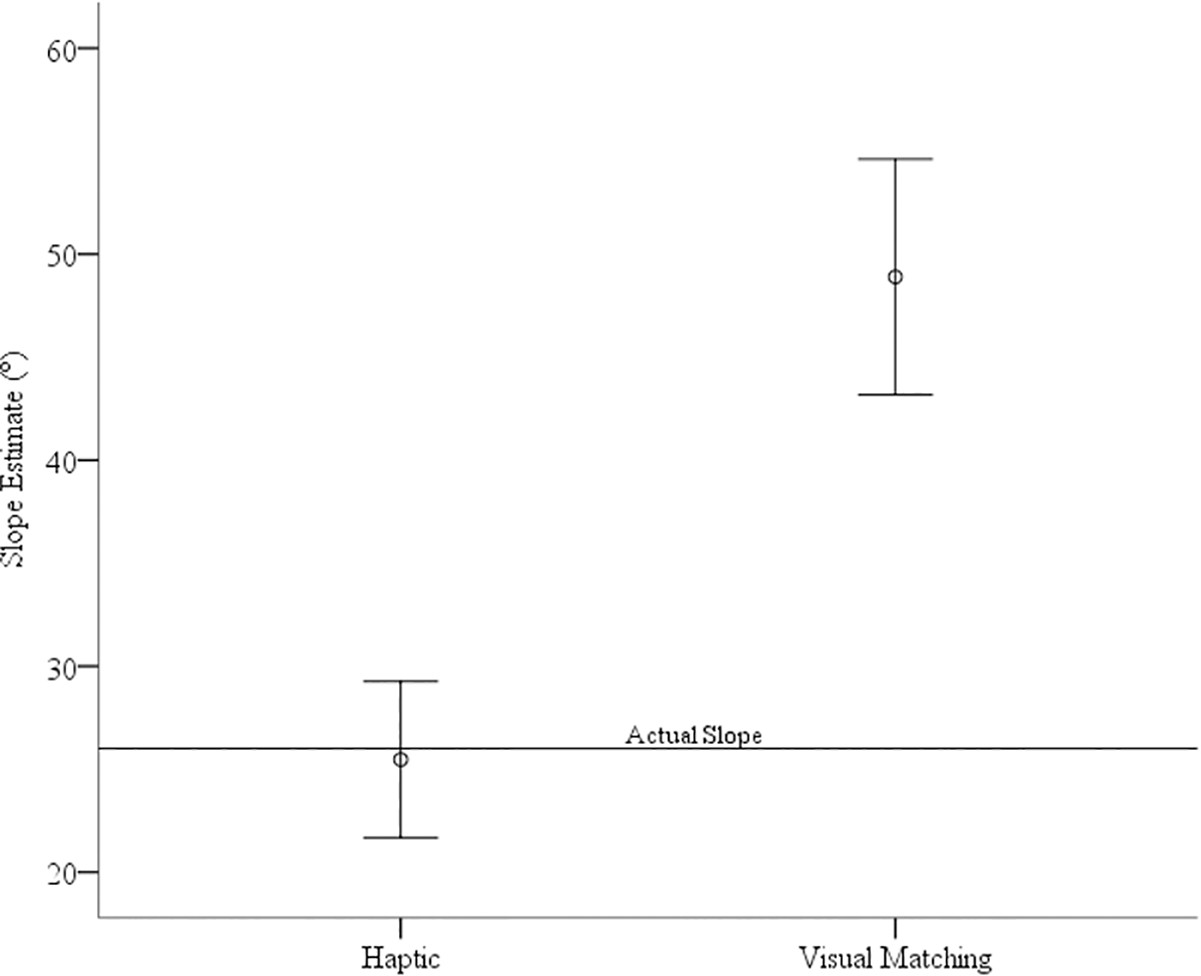
Estimates of slope for the haptic and visual matching tasks. The line represents the actual slope. Error bars represent 95% CIs.

**Fig 9:**
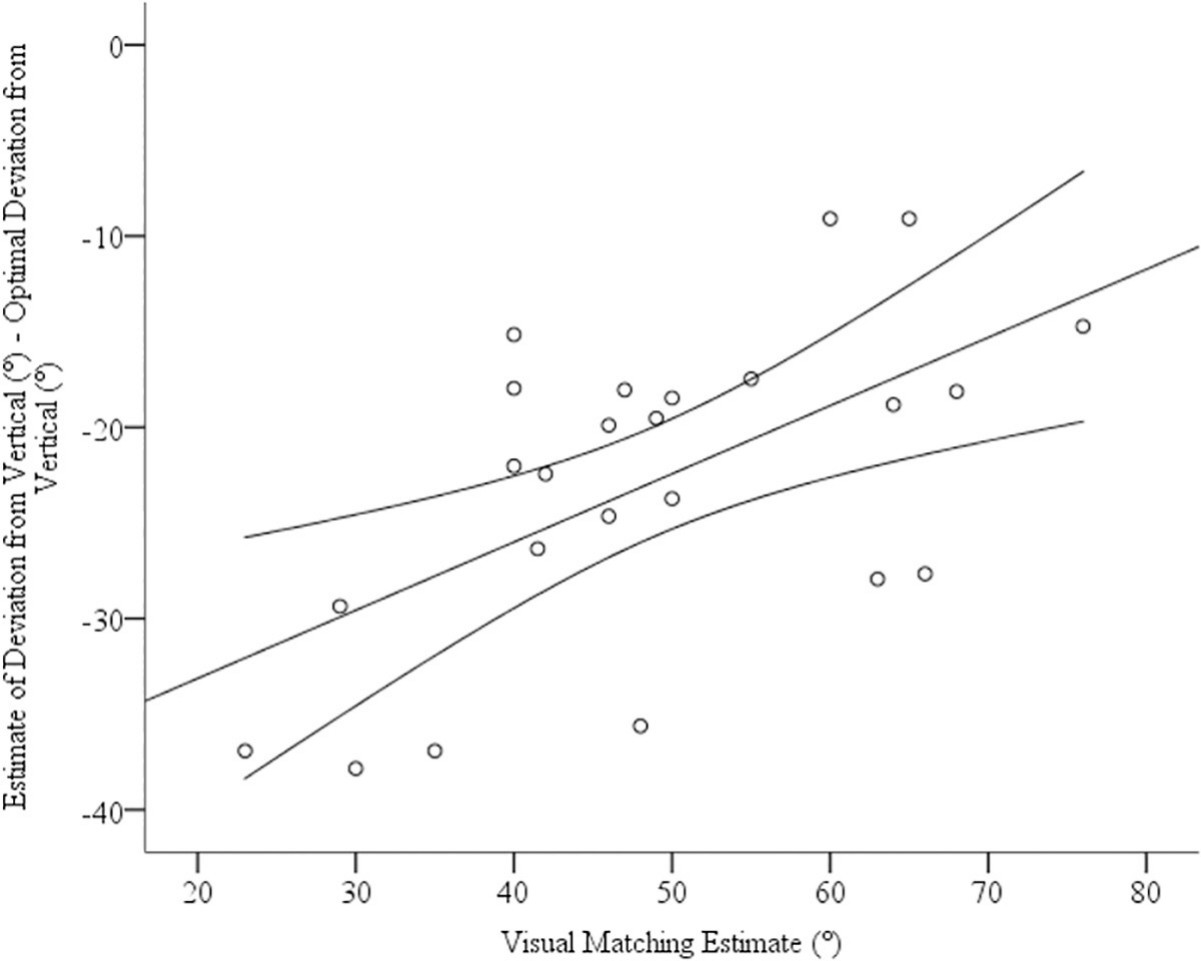
The relationship between route choice and slope estimates. Error bars represent 95% CIs.

To assess judgements of steepness, we compared both the verbal and haptic estimates to accuracy (26°) using one-sample t-tests. Visual matching estimates were greater than the actual incline, *M* = 48.17°, *SE* = 1.72°, *t*(25) = 8.50, *p*<0.001, *d* = 1.67. Haptic estimates were not significantly different than the actual incline, *M* = 24.95°, *SE* = 8.81°, *t*(25) = .61, *p* = 0.55. see Fig 8. We were interested in whether estimates of steepness where related to errors in optimal path choice. Consequently, we conducted correlations between near and far estimates and visual matching estimates of steepness. We found that errors in far estimates were significantly positively correlated to steepness estimates, *r* = 0.59, *p* = 0.001, two-tailed, see Fig 9. In other words, individuals that saw the hills as steeper chose routes that deviated farther from the straight path. We did not find a significant correlation with near estimates, *r* = .26, *p* = .19.

## Experiment 3: Path choice before and after motor experience

Even when hiking, we typically do not choose our own angle of descent, but rather we pick among path that has been pre-specified, either by wear by previous travelers or by proper paths. Thus, in the real world, the choice is usually between different existing paths rather than creating one’s own path. Thus, although individuals may not be able to create the energetically optimal path, they may be able to identify it and chose it when presented amongst less optimal alternatives. To test this contention, we had participants engage in a forced choice task in which they were presented with two paths.

Experiments 1 and 2 have all shown that people do not adjust to the optimal gradient of ascension when ascending hills. People consistently chose paths that are more vertical and direct than paths that are more zig-zagged and require less energy. If people are given motor feedback about the difficulty of hill ascension, perhaps they may adjust their route choice to less energetically expensive route. Thus, here we had participants chose their route choice before and after gaining motor experience ascending an incline.

### Methods

#### Participants

Twenty-two students at Lancaster University volunteered to participate in return for credit in an introductory psychology course. All participants had normal or corrected-to-normal vision and had no morphological abnormalities. All participants provided informed consent and the study was approved by the Lancaster University Ethic Committee.

#### Stimuli and apparatus

**Fig 10:**
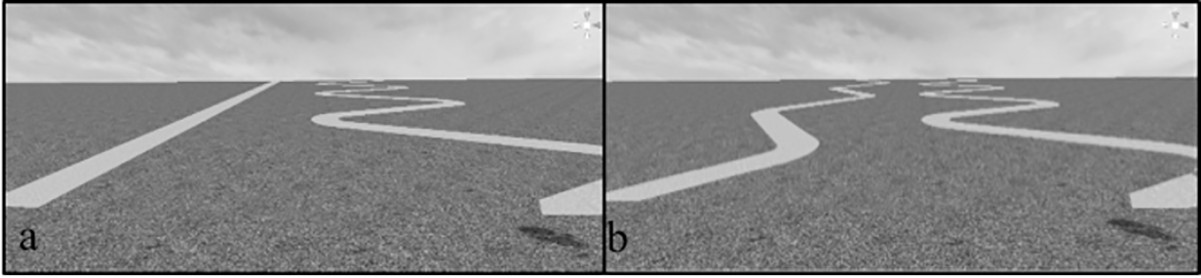
Panel A) An illustration of trials in which participants chose between straight and zigzag paths. For hills below 15°, the optimal path was the straight path; for hills above 15°, the optimal path was the zigzag path. Panel B) An illustration of trials in which participants chose between 2 zigzag paths. For hills below 15°, the optimal path was the less zigzagged path; for hills above 15°, the optimal path was the most zigzagged path.

The virtual environment was presented in an Oculus Rift DK2 head-mounted display (HMD) which provided stereopsis. The virtual environment and experimental program was programmed using Unity3D gaming engine. The participants provided responses using a standard Xbox controller. The environment consisted of a grassy hill. On the hill, we positioned two brick paths as well as a consistent grass texture provide cues to size and depth, see Fig 10. Participants were presented with 8 different hills slants (from 0° to 35° in 5° increments). Hence, 4 hills were above 15° and 4 were above. For 6 of the trials the optimal path was on the left and for the remaining trials, the optimal path was on the right.

For 4 of the trials, participants were presented with a straight path and a large zigzag, see Fig 9A. For 4 other trials, participants were presented with a smaller zigzag and a straight path. For the final four trials, participants were presented with the large zigzag and a small zigzag, see Fig 9B. For hills above 15°, the large zigzag was the optimal zigzag and the small zigzag was half of the optimal zigzag. Hence, we could compare between mere straight biases and alternative less deviations from vertical (lesser zigzags), see Table 1. For hills below 15°, the large zigzag was a 45° deviation from vertical (which would be optimal for the 20°, the smallest optimal deviation from vertical for hills above 15°) and the small zigzag was 22.5° (half of that).

**Table 1:** For each hill, the deviations from vertical in the different trials are listed in degrees.

	Straight Versus Zigzag (°)		Zigzag versus Zigzag(°)	
Hills Below 15°	Optimal	Sub-optimal	Optimal	Sub-optimal
0	0	22/45	22	45
5	0	22/45	22	45
10	0	22/45	22	45
15	0	22/45	22	45
Above 15°				
20	45/22.5	0	45	22.5
25	55/27.5	0	55	27.5
30	61/30.5	0	61	30.5
35	65/32.5	0	65	32.5

Trials were presented in random order. For each hill, participants could choose the optimal route using the rear top triggers on the Xbox controller. The left trigger selected the left path, and the right trigger selected the right path. After either trigger was pressed, a new trial was presented. The experiment consisted a total of 96 trials (8 hill gradients x 12). To provide motor experience, we used a JLL-T100 incline trainer treadmill whose incline can be adjusted from 0° to 21.80° (40% gradient); the treadmill could only be adjusted by increments of 1% gradient.

#### Procedure

Prior to donning the HMD, participants were handed the Xbox controller and instructed how to complete the experiment. They were instructed that they would be standing at the foot of a hill on which there were two paths. Their task was to pick the path on which they would expend the least amount of energy (we clarified this for participants in several ways by explaining that this would be the easiest path on which they would feel the least tired when finished ascending the hill). Participants then donned the HMD and completed the virtual reality program.

After the first completion of the virtual reality program, participants walked on the treadmill for 5 minutes. After each minute on the treadmill, the incline was increased by 10% gradient. Thus, for the first minute, participants walked on a flat treadmill; for the second minute, participants walked on a 10% gradient (5.71°); for the third minute, participants walked on a 20% gradient (11.31°); for the forth minute, participants walked on a 30% gradient (16.70°); and, for the fifth minute, participants walked on a 40% gradient (21.80°). After five minutes, participants were led off the treadmill, put the HMD back on, and completed the virtual reality program a second time. Afterwards, they completed a questionnaire that assessed physical fitness, exercise practices, and frequency of walking and climbing hills.

### Results

In order to assess bioenergetic sensitivity, we calculated the probability of choosing the bioenergetically optimal path choice for each participant in the pre-test and the post-test. Within each test, we calculated the probabilities for the straight versus large zig-zag path, straight versus small zig zag, and large zigzag versus small zigzag for above and below 15°. This transformation led to 12 probabilities per participant.

We then conducted a repeated-measures ANOVA with pre/post, above/under 15°, and path choice (straight/large zigzag, straight/small zigzag and small/large size zag) as within-subjects variables and probability scores as our dependent measure. We found no significant main effects of pre/post, above/under 15°, or path choice, *ps>*0.09. However, we did find significant interactions between pre/post and above/under 15°, *F*(1, 22) = 18.60, *p* < .001, *ŋ*_*p*_^*2*^ = 0.47.

Post-hoc comparisons showed that for hills below 15°, participants were significantly more likely to choose the less optimal path following motor experience, *M* = .57, *SE* = .04, than prior to motor experience, *M =* 0.77, *SE* = .04, *t*(21) = 3.47, *p =* 0.002. Basically, participants were more likely to opt for the zigzag path rather than the straight path. Similarly, for hills above 15°, participants were significantly more likely to choose the more optimal zigzag path following motor experience, *M* = 0.75, *SE* = .06, than prior to motor experience, *M* = 0.53, *SE* = .07, *t*(21) = 4.39, *p*<0.001.

**Fig 11:**
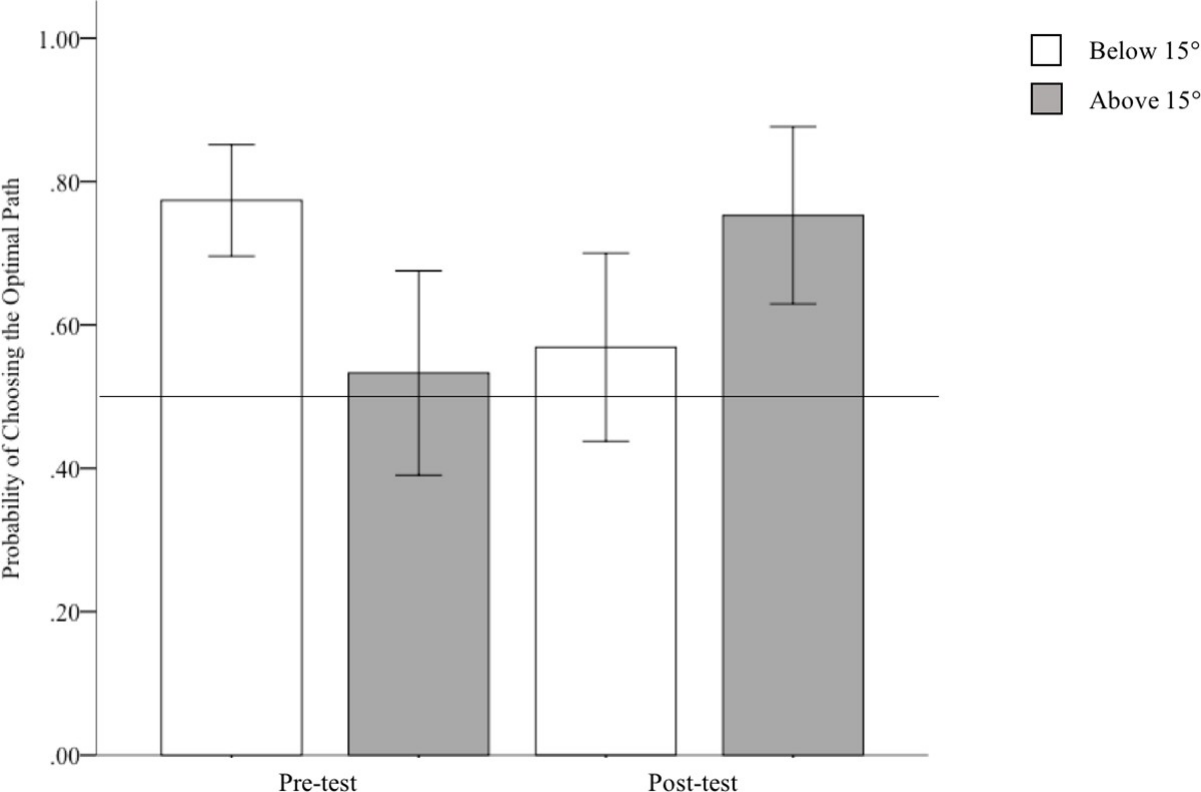
Probability of choosing the optimal route choice for hills above and below 15° before and after motor experience. Error bars represent 95% CIs.

Additionally, we found a significant interaction between above/under 15° and path choice, *F*(2, 42) = 5.52, *p* = 0.007, *ŋ*_*p*_^*2*^ = 0.21, see Fig 11. To investigate this in more detail, we conducted two separate repeated-measures ANOVAs for above and below 15°. Each ANOVA had pre/post and path choice as within-subjects variables and probabilities as the dependent measure. For hills under 15°, we found a significant effect of pre/post (as described above in the post-hoc tests), *p* = 0.002. We also found a significant effect of path choice, *F*(2,42) = 4.25, *p* = 0.02, *ŋ*_*p*_^*2*^ = 0.17. Bonferonni post-hoc tests revealed no differences between the different path choices, *ps >* .10.

For hills above 15°, we found a significant influence of pre/post as reported above, *p* < .001. We also found a significant influence of path choice, *F*(2, 42) = 5.35, *p* = .009, *ŋ*_*p*_^*2*^ = .20. However, Bonferonni post-hoc tests revealed no differences between the different path choices, *ps >* 0.07.

## Discussion

People’s route choices when ascending hills do not indicate a high degree of sensitivity to energetic expenditure. People prefer straighter paths, and consequently, do not take the energetically optimal route both when generating their own path and when picking between different paths in both real and virtual environments. However, we do find that motor experience ascending a slant leads to increases in the probability of choosing routes that deviate more from vertical whether they are energetically efficient or not.

People seem to choose straight, direct paths. One possible reason for this tendency is that, in an environment with abundant resources, time is deemed as more important than energetic expenditure. Although we directly instructed participants to make their route choices based on energetic expenditure, clearly, none of our participants were in any danger of energy depletion. The straight path is the optimal path in terms of time, so perhaps participants saw the straight path as the fastest way up the hill, and the zig-zag paths appeared burdensomely long. Following motor experience, where we emphasized the difficulty of climbing steep hills, participants then began to opt for the more zigzag paths. It is possible this emphasis led participants to weigh energetic expenditure as more important than time.

Another possible reason for choosing the straight path is lack of experience with steep hill ascension. In our environments, we rarely, if ever, climb hills steeper than 14°. For example, as previously mentioned, the steepest paved road in the world is only 19°, and the steepness of this road is such a rare occurrence that it has become an internationally popular tourist destination. In the rare event that we ascend steep hills, we are usually aided by stairs or some other convenience that allows us to avoid climbing directly up the hill. Hence, most people could be unaware of the energetic costs associated with climbing steep hills, and consequentially underestimate their bioenergetic costs. As a result, we opt for shorter routes, because the energy expended with respect to gradient is underestimated relative to energy expended due to length. If this is the case, then individuals from mountainous or less sculpted environments should have greater sensitivity to the bioenergetic costs associated with route choice due to greater experience with hill ascension. In fact, some tribes in Africa still engage in persistence hunting [24]; it would be interesting to see if these individuals show a greater degree of bioenergetic sensitivity to route choice.

A similar reason for this straight path bias is that choosing the straight path is an efficient satisficing heuristic that works for the vast majority of the environmental topographies that we encounter and/or act upon. The cost of the information processing associated with determining the most bioenergetically efficient route choice likely supersedes the costs associated taking a straight path instead of a zigzagged path up a steep hill (on the rare instance in which we ascend one). Hence, we take the straight path, because it almost always is the most efficient route.

Following brief motor experience with hill ascension, participants chose more bioenergetically efficient paths for hills above 15° and less bioenergetically efficient paths below 15°. However, overall, if merely considering both above and below together, after motor experience, participants chose zigzag paths over straight paths regardless of whether it was the optimal path. One could attribute this finding to response bias in that participants gleaned that we expected them to choose more zigzag paths and complied with our hypothesis. We cannot completely rule out this out. However, for participants to have anticipated this hypothesis, they must have found the motor experience with hill ascension more difficult than they anticipated and consequently identified the zigzag path as the easier option. Otherwise, they would have just as likely anticipated that we expected them to increase the number of straight paths that they chose. Additionally, for the 5° and 10° hills, participants were still more likely to take the straight path than the zigzag path, suggesting that they did not merely adopt a strategy of always picking the zigzag path after receiving motor experience. As a result, a more likely explanation of the change after motor experience is that participants underestimated the difficulty associated with ascending steep hills.

We found similar results in participants’ route choices in virtual reality as we found in the real world. This finding corresponds to previous research that has found that hills steepness is perceived similarly in real and virtual environments [22]. We did find smaller errors in the real world in that people did choose a path that had a greater deviation from vertical. However, aside from taking place in the real world, Experiment 2 also differed from Experiments 1 in that participants were required to partially ascend the hill. As shown in Experiment 3, even brief motor experience ascending hills leads to more bioenergetically efficient route choice. Hence, the slight mean difference in deviation from vertical in Experiments 1 and 2 could also be attributed to this design difference. More importantly, in both Experiments 1 and 2, we found large underestimates in the optimal deviation from vertical in people’s path estimates. Consequently, we can be reasonably sure that our results in VR generalize to the real world.

Interestingly, we found that individuals that perceived hills steeper also chose paths that had a greater deviation from vertical. In other words, those that saw the hill as being steeper chose paths required less energy to ascend. A wealth of research has suggested that our perceptions of hill steepness are influenced by our ability to ascend hills [23]. For example, out of shape, elderly and encumbered people perceive hills to be steeper than individuals who are physically fit, youthful and unencumbered [25]. Therefore, our perceptions of hill steepness may influence the paths we choose when ascending hills in that those with less bioenergetic resources see the hill as steeper, and therefore opt to take routes that are more energetically conservative. Conversely, those with more bioenergetic resources see hills as shallower and, therefore take the straighter, shorter paths. However, an alternative explanation could be that fitness influences both estimates independently of each other or even that the type of route that the individual plans to take influences perceptions of steepness. Future research should be able to distinguish between these possibilities.

In our modern, day to day environment, the ability to distinguish between paths of various energetic expenditure is much less important than it was when we relied on long distance running as a method of food procurement. However, we still face situations in which choosing energetically efficient paths would be extremely beneficial, albeit not as routinely. Consider soldiers in the military traversing the landscape in the Middle East or hikers lost in the mountains. The visibly at the top of a hill provides vital information about their surroundings and the location of friend or foe. Additionally, in both of these cases, energy conservation is of upmost importance. These findings suggest that these individuals may expend energy unnecessarily by taking inefficient routes.

These findings also beg the question as to why mountain paths on steep mountains follow more bioenergetically optimal zigzagged paths. We can think of several reasons why this may be the case. Firstly, our population consisted of mainly UK and US undergraduates that have less experience with traversing steep hills than those living in mountainous terrains. Indeed, likely choosing straighter paths is the best heuristic for these students to use in an environment when hills over 15° are rarely encountered. Similarly, perhaps discovering the optimal path is a learning process that occurs over time through trial and error. Likely, the individuals that created these mountain paths climbed these hills often over the course of their life, and eventually adopted the optimal path following a good deal of experience. Our findings in Experiment 3 support this contention in that participants were more likely to choose zigzagged paths on steep hills when provided with motor experience climbing steep hills. However, from our results, we can be reasonably sure that the identification of the bioenergetically optimal route choice is not automatically instilled in us, but rather something that we develop through experience.

This research does not just end at energy expenditure, but can also be applied to other variables associated with traversing inclines. If we are bad at determining bioenergetic costs, it is likely that we are also bad at determining the safety associated with different paths especially for those who are ageing or engaged in physical rehabilitation. By mapping out our capabilities and deficiencies, we will be able to develop heuristics/tools that take advantage of our strengths and compensate for our weaknesses when making decisions with respect to path choice.

## Supporting information

S1 File Experiment 1 data. Data file for Experiment 1.
(SAV)

S2 File Experiment 2 data. Data file for Experiment 2.
(SAV)

S3 File Experiment 3 data. Data file for Experiment 3.
(SAV)
